# Challenging Diagnosis of Basal Cell Carcinoma: A Case Report Emphasizing the Clinical Utility of Dermoscopy

**DOI:** 10.7759/cureus.59274

**Published:** 2024-04-29

**Authors:** Ahmed M Eldaboush, Ashfaq A Marghoob

**Affiliations:** 1 Dermatology, Faculty of Medicine, Al-Azhar University, Cairo, EGY; 2 Dermatology Service, Memorial Sloan Kettering Skin Cancer Center, Hauppauge, USA

**Keywords:** differential diagnosis, dermatoscope, basal cell carcinoma, dermoscope, dermoscopy

## Abstract

Dermatological conditions often present diagnostic challenges due to their diverse manifestations and overlapping clinical features. In such cases, dermoscopy, a non-invasive imaging technique, has emerged as a valuable tool to enhance diagnostic accuracy and guide clinicians in reaching an appropriate differential diagnosis. By visualizing subsurface skin structures and microvascular patterns, dermoscopy provides additional information that complements clinical examination, aiding in the recognition of specific dermatoses and the differentiation between benign and malignant skin lesions. Herein, we present a case that demonstrates the utility of dermoscopy in distinguishing between an initial broad list of differential diagnoses, namely, basal cell carcinoma, squamous cell carcinoma in situ, and other inflammatory dermatitides, such as psoriasis and atopic dermatitis, and narrowing down the differential diagnosis to just one likely diagnosis, which was basal cell carcinoma in our case.

## Introduction

Dermoscopy plays a pivotal role in enhancing diagnostic precision in dermatology. Herein, we illustrate the value of dermoscopy in diagnosing a challenging case of basal cell carcinoma.

## Case presentation

**Figure 1:**
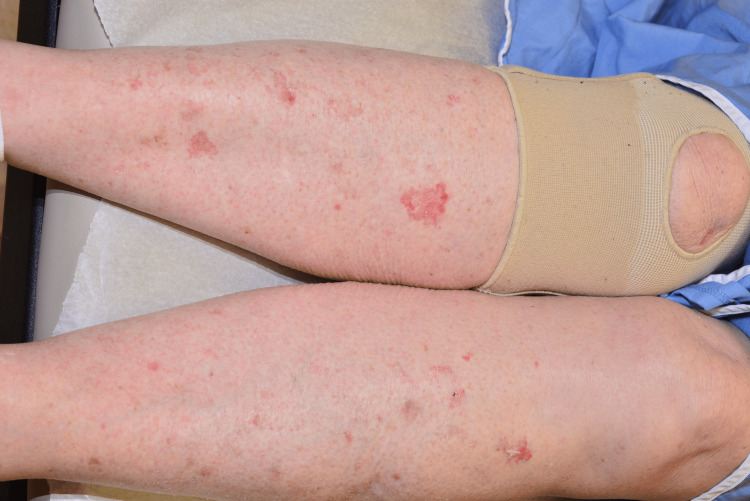
Scattered well-demarcated patches and plaques with a serpiginous border with overlying, fine, adherent scale and focal crusts on the bilateral lower extremities

A female in her 60s presented to the clinic with an asymptomatic scaly rash involving the torso and bilateral lower extremities. The rash was present for a few years and the individual pink areas had slowly enlarged. She had used moisturizers without any improvement and her primary care physician prescribed topical steroids and this did not provide any relief. On examination, there were scattered well-demarcated patches and plaques with a serpiginous border with overlying fine adherent scale and focal crusts (Figure 1).

**Figure 2:**
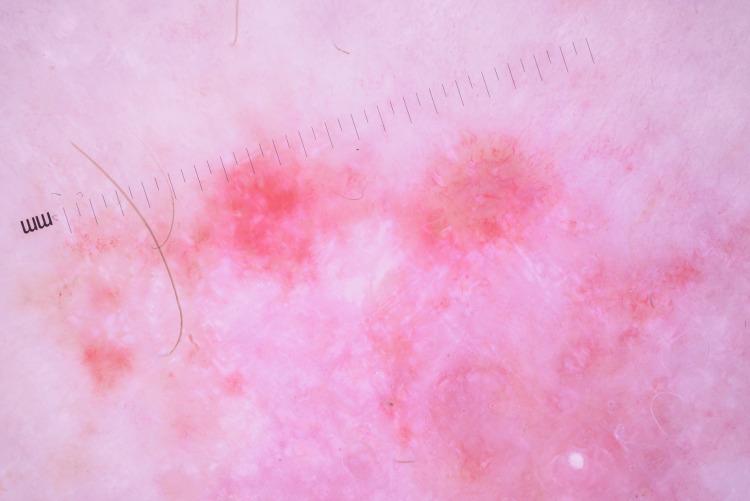
Dermoscopy of one of the lesions showing shiny white structures and thin telangiectatic vessels

The clinical differential diagnosis included inflammatory conditions, such as psoriasis and eczema, and neoplastic conditions including superficial basal cell carcinoma (BCC) and in-situ squamous cell carcinoma (SCCIS). Dermoscopy of the lesions revealed shiny white structures and thin telangiectatic vessels (Figure 2), which, by themselves, are not 100% diagnostic of any specific entity.

**Figure 3:**
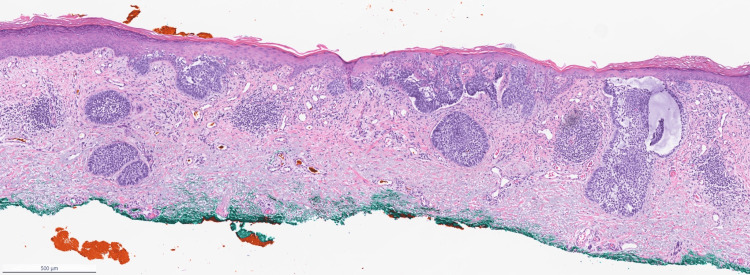
Basal cell carcinoma of the leg Multiple palisading basaloid cell nests can be seen along the dermo-epidermal junction as well as in the superficial dermis. Hematoxylin and eosin staining of a leg lesion. Magnification, x20.

The lesions did not display dotted or glomerular vessels, and no focal yellow clods were visualized on dermoscopy. The dermoscopic findings were not consistent with an inflammatory condition and were also not consistent with SCC. By eliminating psoriasis, eczema, and squamous cell carcinoma from the differential, the diagnosis is now narrowed down to BCC. Armed with this information, the dermoscopy features were re-evaluated and the findings of shiny white structures and thin vessels were deemed most consistent with the diagnosis of superficial BCC. A biopsy of the two lesions on her leg confirmed the diagnosis of multiple superficial and nodular BCCs on histopathology (Figure 3).

## Discussion

Dermoscopy increases the clinician’s diagnostic accuracy [1] and significantly improves the clinician’s benign-to-malignant biopsy ratio [2]. However, what studies do not adequately capture is the value of dermoscopy in narrowing the clinical differential diagnosis, which, combined with the clinical-dermoscopy correlation, can provide a final diagnosis through a process of elimination or default diagnosis.

Dermoscopic structures could potentially be diagnostic for specific disease entities such as leaf-like areas for basal cell carcinoma (BCC). Furthermore, there are also dermoscopic structures that are suggestive of but not diagnostic of specific disease entities such as shiny white structures seen in cutaneous malignancies. Both the presence as well as the absence of dermoscopic structures can support or negate disease entities in the clinician’s differential diagnosis. Dermoscopy of psoriasis would reveal dotted vessels/red globules distributed homogeneously throughout the lesions in nearly all psoriatic plaques, a finding not seen in our case [3]. Atopic dermatitis (eczema), similar to psoriasis, can also display dotted vessels and scale; however, due to spongiosis, it is common also to see yellow clods, corresponding to serum exudate in eczema [4]. The lack of dotted vessels and yellow clods on dermoscopy excludes inflammatory conditions from the differential diagnosis. Furthermore, the lack of an orange color in the lesions makes a granulomatous or lichenoid process unlikely [5]. With this information, the differential diagnosis narrowed to a likely neoplastic process with superficial BCC as the leading suspect, but cutaneous T-cell lymphoma (CTCL) was also considered. As already stated, the lesions did not reveal glomerular vessels nor focal scale crust, thus making the diagnosis of squamous cell carcinoma in situ (SCCIS) highly unlikely [6]. CTCL, characterized by fine short linear vessels, orange to yellow patchy areas, and a distinct vascular structure composed of a dotted and short, curved linear vessel that resembles spermatozoa [7], were not seen.

The default leading diagnosis became superficial BCC, even though multiple similar lesions were seen. The non-pigmented subtype is associated with short, fine telangiectasia, small erosions, and shiny white structures in the form of blotches and strands [8]. Dermoscopy indeed revealed shiny white structures and short, fine superficial telangiectasia.

Histopathology from a lesion on the patient's leg revealed that multiple palisading basaloid cell nests can be seen along the dermo-epidermal junction and in the superficial dermis, consistent with BCC, which corroborated the dermoscopy findings and confirmed the final diagnosis of multiple superficial and nodular BCCs.

## Conclusions

In summary, evaluating for the presence or absence of dermoscopic structures can assist the clinician in excluding disease entities from the clinical differential diagnosis, often leaving one or two disease entities in the final differential. At this point, the clinical-dermoscopy correlation of the structures present can usually confirm the diagnosis of one disease, which in our case was superficial and nodular BCCs.

## References

[1] Vestergaard ME, Macaskill P, Holt PE, Menzies SW (2008). Dermoscopy compared with naked eye examination for the diagnosis of primary melanoma: a meta-analysis of studies performed in a clinical setting. Br J Dermatol.

[2] Argenziano G, Cerroni L, Zalaudek I (2012). Accuracy in melanoma detection: a 10-year multicenter survey. J Am Acad Dermatol.

[3] Lallas A, Kyrgidis A, Tzellos TG (2012). Accuracy of dermoscopic criteria for the diagnosis of psoriasis, dermatitis, lichen planus and pityriasis rosea. Br J Dermatol.

[4] Navarini AA, Feldmeyer L, Töndury B, Fritsche P, Kamarashev J, French LE, Braun RP (2011). The yellow clod sign. Arch Dermatol.

[5] Bañuls J, Arribas P, Berbegal L, DeLeón FJ, Francés L, Zaballos P (2015). Yellow and orange in cutaneous lesions: clinical and dermoscopic data. J Eur Acad Dermatol Venereol.

[6] Bugatti L, Filosa G, De Angelis R (2004). Dermoscopic observation of Bowen's disease. J Eur Acad Dermatol Venereol.

[7] Lallas A, Apalla Z, Lefaki I (2013). Dermoscopy of early stage mycosis fungoides. J Eur Acad Dermatol Venereol.

[8] Scalvenzi M, Lembo S, Francia MG, Balato A (2008). Dermoscopic patterns of superficial basal cell carcinoma. Int J Dermatol.

